# Methylglyoxal (MG) and Cerebro-Renal Interaction: Does Long-Term Orally Administered MG Cause Cognitive Impairment in Normal Sprague-Dawley Rats?

**DOI:** 10.3390/toxins6010254

**Published:** 2014-01-07

**Authors:** Kimio Watanabe, Kana Okada, Ryoji Fukabori, Yoshimitsu Hayashi, Koichi Asahi, Hiroyuki Terawaki, Kazuto Kobayashi, Tsuyoshi Watanabe, Masaaki Nakayama

**Affiliations:** 1 Department of Nephrology, Hypertension, Diabetology, Endocrinology and Metabolism, School of Medicine, Fukushima Medical University, 1 Hikarigaoka, Fukushima 960-1295, Japan; E-Mails: caco@fmu.ac.jp (Y.H.); asahi@fmu.ac.jp (K.A.); terawaki@fmu.ac.jp (H.T.); twat0423@fmu.ac.jp (T.W.); masanaka@fmu.ac.jp (M.N.); 2 Graduate School of Integrated Arts and Sciences, Hiroshima University, 1-7-1 Kagamiyama, Higashi-Hiroshima 739-8521, Japan; E-Mail: kaokada@hiroshima-u.ac.jp; 3 Department of Molecular Genetics, Institute of Biomedical Sciences, School of Medicine, Fukushima Medical University, 1 Hikarigaoka, Fukushima 960-1295, Japan; E-Mails: fukabori@fmu.ac.jp (R.F.); kazuto@fmu.ac.jp (K.K.)

**Keywords:** cerebro-renal interaction, methylglyoxal, cognitive impairment, chronic kidney disease

## Abstract

Methylglyoxal (MG), one of the uremic toxins, is a highly reactive alpha-dicarbonyl compound. Recent clinical studies have demonstrated the close associations of cognitive impairment (CI) with plasma MG levels and presence of kidney dysfunction. Therefore, the present study aims to examine whether MG is a direct causative substance for CI development. Eight-week-old male Sprague-Dawley (SD) rats were divided into two groups: control (*n* = 9) and MG group (*n* = 10; 0.5% MG in drinking water), and fed a normal diet for 12 months. Cognitive function was evaluated by two behavioral tests (object exploration test and radial-arm maze test) in early (4–6 months of age) and late phase (7–12 months of age). Serum MG was significantly elevated in the MG group (495.8 ± 38.1 *vs.* 244.8 ± 28.2 nM; *p* < 0.001) at the end of study. The groups did not differ in cognitive function during the course of study. No time-course differences were found in oxidative stress markers between the two groups, while, antioxidants such as glutathione peroxidase and superoxide dismutase activities were significantly increased in the MG group compared to the control. Long-term MG administration to rats with normal kidney function did not cause CI. A counter-balanced activation of the systemic anti-oxidant system may offset the toxicity of MG in this model. Pathogenetic significance of MG for CI requires further investigation.

## 1. Introduction

Methylglyoxal (MG) is a highly reactive alpha-dicarbonyl and protein-bound type compound with a molecular weight of 72 dalton [1]. MG binds to and modifies arginine, lysine, and cysteine residues, which leads to production of a variety of advanced glycation end products (AGEs) [2]. 

Accumulative clinical evidence has revealed that cognitive impairment (CI) is commonly prevalent among patients with a various stages of chronic kidney disease (CKD), ranging from early to advanced stage, including cases on dialysis treatment [3,4,5,6,7]. Those findings strongly indicate a possible involvement of an unknown causative factor, which is connected with the uremic status of CKD. Interestingly, recent clinical studies have reported that higher levels of serum MG or MG modified protein were associated with faster cognitive decline [8] and with poorer memory, poorer executive function and lower grey matter volume in the general population [9]. Thornalley PL, *et al.* reported that AGEs in cerebrospinal fluid were associated with CI [10]. MG is one of the uremic toxins that enhance oxidative stress, and its plasma level is increased according to the level of kidney dysfunction [11]. Taken together, this indicates that MG plays a crucial role in the progression or development of CI.

It is still unknown whether MG is a direct causative substance of CI, including whether it plays a role in the pathology and mechanism of CI. Although previous researchers have demonstrated MG-induced cytotoxicity in cultured hippocampal neurons and cerebral cortex neuronal cells [12,13], it is unclear whether the same effect occurs in living animals. 

The aim of the present study is to confirm whether long-term orally administered MG induces CI in Sprague-Dawley (SD) rats with normal kidney function. 

## 2. Results

### 2.1. Physical Findings and Laboratory Tests

Body weight, mean blood pressure, uric protein, renal function and blood glucose did not show any statistical differences between the groups through the two phases (Table 1 and Table 2). The mean serum MG level was significantly increased in the MG group when MG was continuously administrated orally during a period of 39 weeks (from age of 8 to 47 weeks): MG *vs.* control, 495.8 ± 38.1 *vs.* 244.8 ± 28.2 nM (*p* < 0.001) (Table 2). The drinking volume measured at 15 week of age did not differ between the group: Control *vs.* MG, 17.0 ± 8.9 *vs.* 19.1 ± 6.9 mL/day.

**Table 1:** Timeline chart of the experiments and physical findings.

Phase	Age in weeks (W)	MG exposure (W)	Behavior test	Urine & blood test	Mean BP (mmHg)		*p* Value	Body weight (g)		*p* Value
					Control (*n* = 9)	MG (*n* = 10)		Control (*n* = 9)	MG (*n* = 10)	
**Phase I**	8	START	-	-	101 ± 2.1	105 ± 2.0	0.221	281 ± 3.3	277 ± 3.8	0.527
	16	+8	-	Urine Test (1)	99.0 ± 4.0	99.0 ± 3.3	0.935	506 ± 10.5	487 ± 10.7	0.238
	16	+8	Object exploration Test (1)	-	-	-	-	-	-	-
	20 ~ 23	+12~+15	Radial-arm Maze Test (1)	-	82.0 ± 2.1	79.7 ± 3.4	0.594	533 ± 10.3	509 ± 11.9	0.159
**Phase II**	31	+23	Object exploration Test (2)	-	-	-	-	569 ± 11.3	539 ± 12.8	0.116
	42 ~ 44	+34~+36	Radial-arm Maze Test (2)	-	-	-	-	-	-	-
	45	+37	-	Urine Test (2)	-	-	-	-	-	-
	47	+39	(Sacrificed)	Blood Test	93.4 ± 3.3	94.2 ± 4.5	0.674	603 ± 14.2	566 ± 14.5	0.083

Notes: MG: Methylglyoxal; BP: blood pressure.

**Table 2:** Renal function and oxidative stress marker tested in each phase.

Parameters	Phase I			Phase II		
	Control (*n* = 9)	MG (*n* = 10)	*p* Value	Control (*n* = 9)	MG (*n* = 10)	*p* Value
Uric Protein (g/gCrea)	0.7 ± 0.2	1.0 ± 0.5	0.068	3.0 ± 0.7	1.8 ± 0.6	0.201
Urinary 8-OHdG (ng/day)	252.2 ± 21.1	218.9 ± 14.5	0.356	211.9 ± 12.0	205.0 ± 16.3	0.549
Urinary MDA (nmol/day)	106.8 ± 9.2	101.3 ± 12.6	0.733	27.0 ± 4.9	27.7 ± 4.8	0.927
BUN (mg/dL)	-	-	-	20.7 ± 0.6	21.9 ± 0.8	0.241
Serum Creatinine (mg/dL)	-	-	-	0.30 ± 0.01	0.33 ± 0.02	0.210
Plasma Glucose (mg/dL)	-	-	-	236.3 ± 7.1	221.9 ± 6.4	0.149
Serum MG (nM)	-	-	-	244.8 ± 28.2	495.8 ± 38.1	<0.001
Plasma MDA (µM)	-	-	-	0.16 ± 0.04	0.14 ± 0.01	0.447
Plasma AGT (ng/mL)	-	-	-	3.2 ± 0.3	2.1 ± 0.1	0.014
Urinary AGT (ng/day)				29.3 ± 6.5	16.3 ± 6.3	0.034
Kidney GPx (mU/mg)	-	-	-	3.6 ± 0.4	5.1 ± 0.4	0.036
Kidney SOD (U/mL/mg)	-	-	-	3.7 ± 0.9	6.3 ± 0.9	0.069

### 2.2. Results of Behavioral Testing

**Figure 1:**
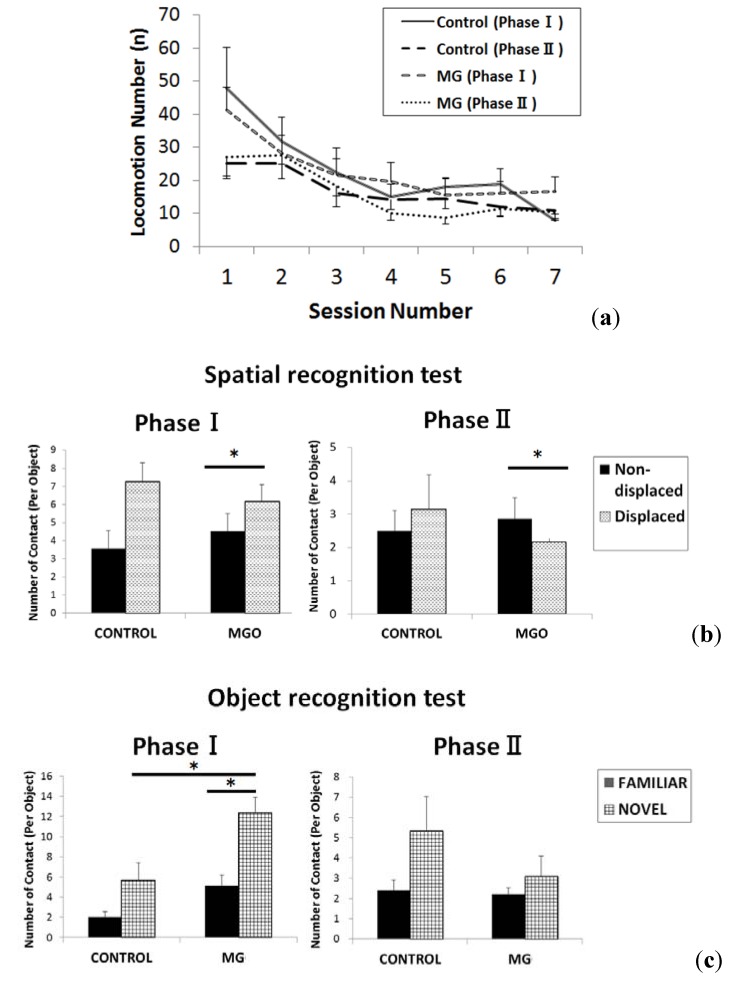
Effect of methylglyoxal (MG) on cognition as tested by the open field test (Object exploration test). Groups of rats were treated with tap water (Control, *n* = 9) or methylglyoxal (MG, 0.5% MG solution as drinking water, *n* = 10) for 39 weeks. Spatial memory was tested at 16 weeks (Phase I) and 31 weeks (Phase II) of age during treatment by an open field test as described in the methods section. (**a**) Locomotion number through all sessions of object exploration test; (**b**) non-displaced *vs.* displaced objects to indicate spatial recognition in object exploration test; (**c**) contact with familiar and new objects in object exploration test. * *p* < 0.05.

The results of object exploration tests in both phases are shown in Figure 1. The locomotion number throughout all sessions in Phase I (16 week of age) and Phase II (31 weeks of age) is shown in Figure 1a. Although there was no statistical difference between sessions, the locomotion number in both groups tended to be smaller in Phase II compared to Phase I. The results of spatial recognition memory, which was tested in Sessions 5 and 6 in Phase I and II, are shown in Figure 1b. In Phase I, the contact number with displaced objects in the MG group was significantly higher compared to that with non-displaced objects, however, in Phase II, it decreased significantly compared to non-displaced objects. There was no statistically significant difference between the two phases in the spatial recognition test in the control group. The results of the object recognition function, which was tested in Session 7 in Phase I and II, are shown in Figure 1c. In Phase I, the contact number with the new object (Novel) in the MG group was significantly higher compared to that with familiar objects and compared to the contact number with the new object in the control group. There was no statistically significant difference in Phase II. 

**Figure 2:**
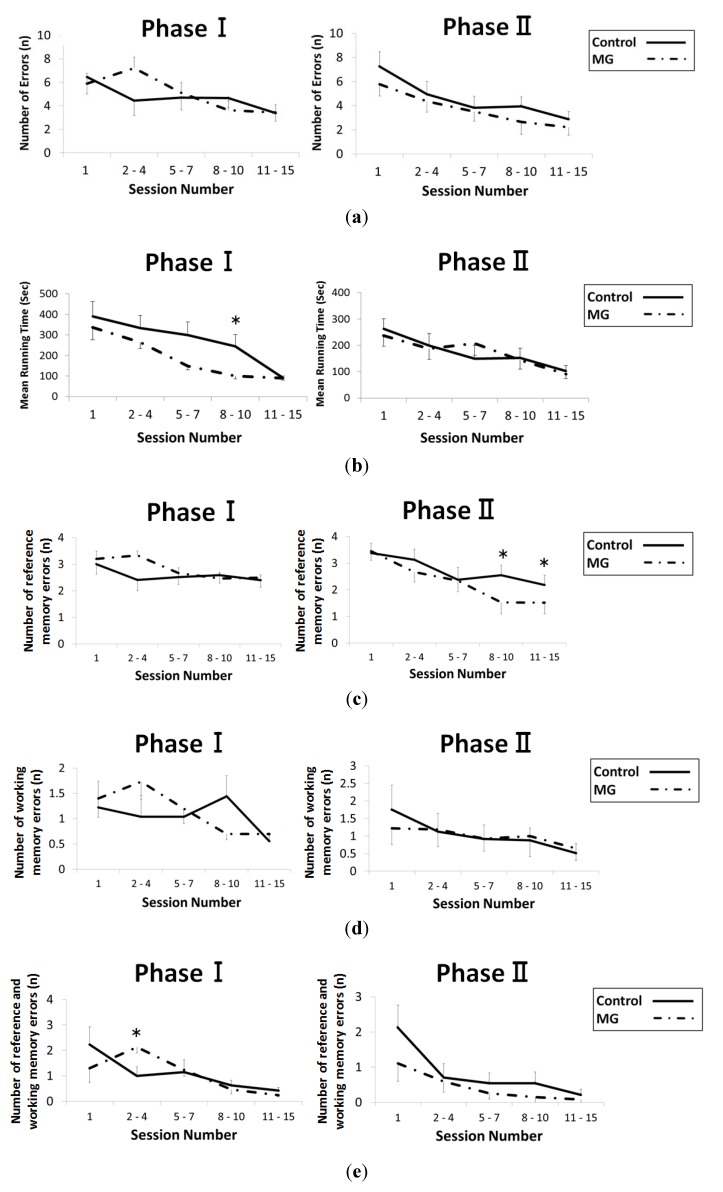
Effect of methylglyoxal (MG) on cognition as tested by the radial-arm maze test. Spatial reference and spatial working memory were tested between 20 and 23 weeks of age (Phase I), and 42 and 44 weeks (Phase II) of age during testing with the radial-arm maze test as described in the methods section. (**a**) The number of total errors in the radial-arm maze test; (**b**) mean running time in the radial-arm maze test; (**c**) number of reference memory errors in the radial-arm maze test; (**d**) number of working memory errors in the radial-arm maze test; and (**e**) number of reference and working memory errors in the radial-arm maze test. * *p* < 0.05.

We examined the radial-arm maze test between ages of 20 and 23 weeks (Phase I), and between the ages of 42 and 44 weeks (Phase II). The results of the maze test, which was conducted to examine the spatial reference memory and spatial working memory, are shown in Figure 2a–e. In both phases, the number of total errors did not reach any statistically significant differences between the groups (Figure 2a). The mean running time in both group in the two phases is shown in Figure 2b. The running time of the control group in Phase I in Sessions 8–10 was significantly higher compared to that of the MG group, however, there was no differences within both groups in Phase II. The number of reference memory errors (entering into arms without rewards) during each Phase is shown in Figure 2c. In Phase I, there was no statistical difference, and in Phase II, there was a significant difference in Sessions 8–10 (*p* = 0.001) and in Session 11–15 (*p* = 0.017). The number of working memory errors (entering an arm containing a reward repeatedly after already finding the rewards) during each Phase is shown in Figure 2d. There was no statistically significant difference between the groups throughout the sessions. The number of reference and working memory errors (repeatedly entering into arms without rewards) during each phase is shown in Figure 2e. Apart from one point (Phase I, Session 2–4), there was no statistically significant difference.

### 2.3. Histological Findings of Brain and Kidney

**Figure 3:**
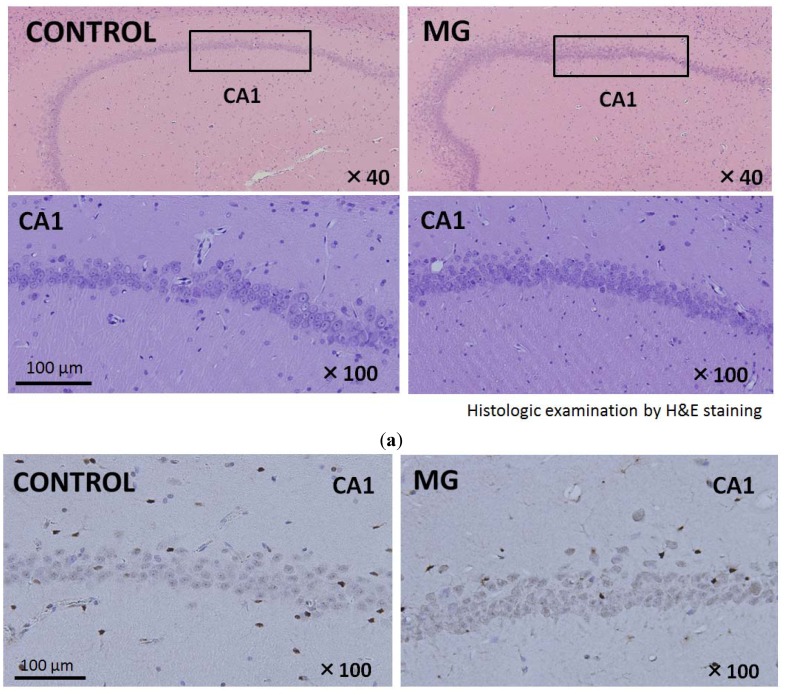
Histological findings at sacrifice (47 weeks of age) in each group. (**a**) Hematoxylin and eosin (H&E)-stained brain sections in the hippocampus region. Neuronal cell damage was not detected in the MG group; (**b**) Caspase-3 staining in the hippocampus region. Apoptotic cells could not be detected in either of the two groups.

The central nervous system (CNS) shows vulnerability to toxic compounds such as MG. Accumulation of MG in the hippocampus has an important role in learning impairment and memory disorders [14]. CA1 and CA3 of the hippocampus and cerebral cortex show particular vulnerability to oxidative stress and are associated with memory and learning [12,13,15]. We examined hematoxylin and eosin (H&E)-stained brain sections. No evidence of neuronal damage was found in the Cornu ammonis 1 (CA1) and 3 (CA3) or in the cerebral cortex. Histological findings of brain tissue in the CA1 region and its surrounding area in each group are shown in Figure 3. Apoptotic cells could not be detected by caspase-3 staining in either group (Figure 3b). From these histological findings, it was judged that there were no alterations in cellular morphology or apoptosis in the hippocampal neuronal cells in either group. There were no differences in terms of percentage of glomerular sclerosis, degrees of tubular atrophy, and degrees of inflammatory cells infiltration from a quantitative perspective among the groups.

### 2.4. Analysis of Oxidative Stress, Antioxidants and Angiotensinogen

We evaluated biomarkers using urine, blood and kidney samples (Table 2). Urinary biomarker of 8-OHdG and MDA, which we tested as oxidative stress marker, and uric protein, which we tested as a marker of renal impairment, were examined in both phases. These markers did not show any statistically significant differences between phases. MDA was also examined by blood sample in Phase II, however, there were no differences between the groups. Renal glutathione peroxidase (GPx) activity was significantly elevated in the MG-treated group (MG *vs.* the control, 5.1 ± 0.4 *vs.* 3.6 ± 0.4 mU/mg (*p* = 0.036)). Superoxide dismutase (SOD) activity tended to be higher in the MG-treated group (MG *vs.* control, 6.3 ± 0.9 *vs.* 3.7 ± 0.9 U/mL/mg (*p* = 0.069)). Urinary and plasma angiotensinogen (AGT) levels were significantly lower in the MG-treated group: MG *vs.* control, 2.1 ± 0.2 *vs.* 3.2 ± 0.3 ng/mL (*p* = 0.014), 16.3 ± 6.7 *vs.* 29.3 ± 6.9 ng/day (*p* = 0.034), respectively. These results are shown in Table 2. 

## 3. Discussion

The present study aims to examine whether MG is a direct causative substance for CI development.

Eight-week-old male SD rats were given MG by *ad lib* drinking water for up to 12 months. We examined the cognitive function using two behavioral tests (object exploration test and radial maze test) in an early (4–6 months of age) and a late phase (7–12 months of age). Although serum MG was significantly elevated in the MG group, the groups did not differ in cognitive function during the course of study, and no histological changes were observed at the end of study. No time-course differences were found for the oxidative stress marker, while antioxidants were significantly increased in the MG group compared to the control.

Before we discuss our speculations of the results, we need to describe some significant limitations of our study. (1) We selected SD rats with normal kidney function; and (2) we could not confirm the MG status in the CNS. As to the first limitation, a kidney injury model might represent very different responses to long-term low doses of oral administered MG, because the interaction of MG with other uremic toxins or with oxidative stress is anticipated in those models. It might also be possible to detect the pathogenesis of the relationship between CI and MG by using diabetes-model animals. As to the second limitation, we failed to examine the MG status in the CNS. The serum MG level was significantly increased in the MG group rats, however, the impact of MG on the CNS was unclear, because we could not detect how much MG passed through the blood brain barrier and reached the CNS. This suggests that a significant amount of MG is thought to be needed in order to enter the brain from the blood. Additionally, in terms of evaluation of the MG status in the CNS, measuring brain and kidney MG levels or MG-induced AGEs (such as *N*-carboxyethyllysine) is thought to be useful.

There are several studies, which examined the effect of an exogenous MG load, which induced various dysfunctions such as renal problems (urinary albumin excretion, glomerular sclerosis and tubular damage), blood pressure elevation, and diabetic-like pathological changes [16,17,18]. In our pilot study, we confirmed that administration of >1% MG to rats caused significant loss of body weight and activity due to its toxicity, so we employed a 0.5% MG solution. Serum MG was significantly elevated in the MG group, and we confirmed that it was possible to mimic the MG status in uremic patients by orally administering MG. We conducted some behavior tests for consideration of CI. Open field tests and radial-arm maze tests have been established as effective procedures for evaluation of locomotor activity, spatial and object recognition memory, and many experiments using these methods have been reported using toxic compounds, including endogenous agents, in rodents [19,20,21,22,23,24,25]. We considered cognitive functioning in two phases: Phase I and II. However, the groups did not differ in cognitive functioning in both phases. Taken together, we concluded that there was no obvious effect of MG within the range of a two-fold increase in serum MG from basal level in the behavior tests. 

Cognitive impairment was not demonstrated by MG loading in this study. One of the acceptable reasons for our results is the MG concentration. The plasma MG levels were thought to be relatively low to induce neuronal damage. Demonstrating the CI based on the orally available dose of MG may be difficult. A high concentration of MG (for example, 100 µM or higher) has been employed in previous studies using cultured hippocampal neurons [15,26]. Additionally, neuronal cell apoptosis has been induced by 2.5 mM of MG exposure in neonatal rat brains [27]. However, serum MG levels in this study were at the nanomole level, and administration of high concentrations of MG was impossible because of the severe inhibition of food intake, which would have led to a fatal condition. This suggests that MG might not actually be a causative factor for the development of CI. Although an effect of MG on the development of CI has been shown in some previous studies [8,9], it might simply be a surrogate marker for pathological condition of CI. Based on the theory, specific conditions, which increase MG levels or accelerate MG toxicity, are thought to be more critical factors. 

Nevertheless, it is not possible to exclude the possibility of MG as a candidate substance for CI based only on our limited data. MG is an uremic toxin, which induces and/or amplifies oxidative stress [28,29,30]. Nakayama *et al* reported that MG reacted characteristically with hydrogen peroxide, and that methyl- and carbon-centered radicals were generated non-enzymatically [30]. In the study, they used MG and hydrogen peroxide in non-toxic concentrations, nevertheless high levels of free radicals were generated. This suggests that the manifestation of MG toxicity is related to the acceleration of oxidative stress, which is induced by MG itself, uremic toxins, aging or diabetes. The animal species might be related to the impact of MG on cognitive function. We used SD rats in the study. Oxidative stress levels in the body are thought to be different between the rat strains, and the Dahl rat was selected in a previous report, which demonstrated renal dysfunction by MG administration [18]. Consideration of MG effects in situations with a disrupted antioxidant system will be an issue in the future. 

The activation of the antioxidant system observed in our protocol is interesting. We speculate that a counter regulation against MG exposure up-regulated the antioxidant system. This phenomenon is sometimes described as “hormesis effect”. Hormesis is the phenomenon by which an organism shows a favorable biological response to low-dose exposures to stressors such as toxic compounds and radiation [31]. The hormetic mechanism responds to oxidative stress by involving an enhanced antioxidant defense, generating endogenous scavengers, and producing enzymes of detoxification [32,33,34,35,36]. The results of the present study in theory consistent with this mechanism.

## 4. Experimental Section

### 4.1. Animals

Young adult male SD rats at 8 weeks of age (SLC, Shizuoka, Japan) were housed under controlled environmental conditions (temperature 22 ± 1.5 °C; humidity 55% ± 5%; dark:light set at 12 h:12 h, lights on at 7 a.m.) with free access to water and standard pellet food. All procedures were conducted in accordance with the National Institutes of Health Guide for the Care and Use of Laboratory Animals, and the study protocols were approved by the animal committee of Fukushima Medical University (approval number: 25026). 

### 4.2. Treatment Protocol

The rats were divided into two groups: the control group (*n* = 9; tap drinking water), and the MG group (*n* = 10; 0.5% grams per volume MG solution (= 69 mM) as drinking water). The rats were fed with standard pellet food (0.8% NaCl; Nihon CLEA Japan, Inc., Tokyo, Japan). Body weight was measured at 8, 16, 23, 31 and 47 weeks of age, and blood pressure and heart rate were measured at 8, 16, 20 and 47 weeks of age by an indirect tail-cuff method (Softron BP-2000; Tokyo, Japan). Timeline chart of the experiments and physical findings are shown in Table 1. We defined the period between the start of protocol (8 weeks of age) and 6 months of age as “Phase I”, and that between 7 months of age and 12 months of age as “Phase II” for descriptive purposes. 

### 4.3. Behavioral Procedure

We conducted two behavioral tests in each phase: (1) object exploration test for assessing spatial memory; and (2) eight-arm radial maze test for assessing spatial memory-related reference and working memory.

**Figure 4:**
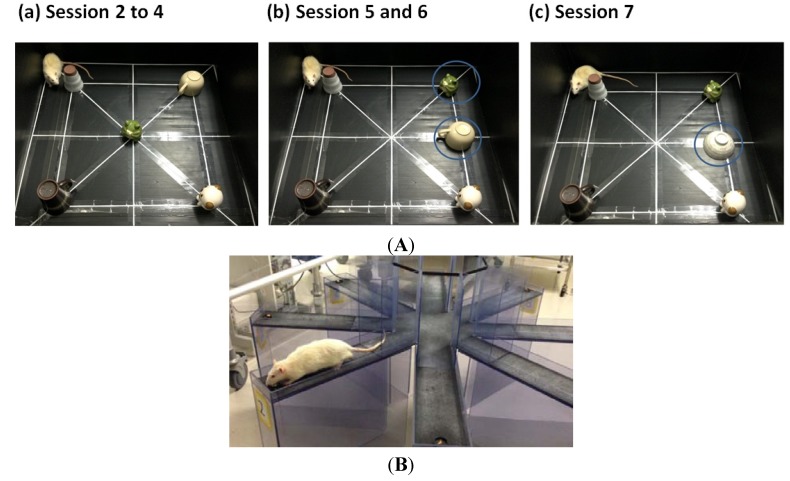
(**A**) Object exploration test. We used the experimental device shown in Figure 4A. We investigated the exploration of five objects in an open field, habituation of locomotion and object investigation, and response to spatial and non-spatial change of rats in each phase. (**a**) The open field and the objects during Sessions 2–4 (habituation); (**b**) The spatial change situation involving the displacement of two objects in Sessions 5 and 6; (**c**) Only one object change situation in Session 7; (**B**) The radial-arm maze test. Cereal as a reward was placed in four of the eight arms. The reward arms were set through the protocol and were different for each animal. We used the experimental device in Figure 4B. We investigated the total number of errors and mean running time of all rats through the sessions in each phase.

#### 4.3.1. Object Exploration Test

Object exploration test was examined at 16 (Phase I) and 31 weeks of age (Phase II). The device and situation of the test are shown in Figure 4A. The device was a square open field, with a side length of 90 cm and a wall height of 45 cm. As seen in Figure 4A, we selected six objects, such as a cup or a doll, which were different in color or shape. The test was divided into seven consecutive 6-min sessions, each of which was separated by a 3-min delay. We divided the open field into 16 compartments and recorded locomotion number as an index of activity and habituation (Figure 1A). Session 1 was a familiarization phase, rats were placed into the empty open field. During Session 2–4, the objects were placed as in Figure 4Aa, and we assessed the habituation by comparing the contact number with the objects through the sessions. In Session 5 and 6, we examined the response to the spatial change by moving two objects (Figure 4Ab). Then, we calculated and compared the contact number of displaced and non-displaced objects. In Session 7, we examined the response to a new object (Novel) by replacing one of the familiar objects with a new one (Figure 4Ac). We also calculated and compared the contact number of familiar and new objects. Three rats (No. 4, 7, 9 in the control group) in Phase I were excluded from the analysis, because they did not move at all in the open field. We conducted the whole test protocol in reference to previous research [37].

#### 4.3.2. Radial Arm Maze Test

Next, we examined the spatial working and reference memory using the radial-arm maze test based on the protocol reported previously [38]. The weight of each animal was restricted and maintained at about 90% (85%–95%) of baseline by limiting food during the examination period. The maze device was elevated about 50 cm above the floor and had a center platform with eight arms radiating from the center (Figure 4B). Holes at the distal end of each arm served as wells for the cereal. The positions of the table, chair, light equipment and experimental device were fixed in the room where the maze was located and served as spatial clues for the animals during the examination period. At the start of each trial, each rat was placed at the center of the platform. Four of the arms contained cereal, and the other four arms did not. The animals remained on the maze until all four rewards were eaten or 10 min had elapsed. The choice of arms and total running times were recorded. The actual time spent on the maze test was recorded as “running time”. If a rat could not find all the rewards in each session, running time was recorded as 600 s. Each rat was tested once per day, and a total of 15 trials was conducted. The errors could be classified mainly into the following three types: (1) Reference memory error: The animal entered an arm without a reward, which meant that the rat did not remember which arms contained the rewards; (2) Working memory error: The animal entered an arm containing a reward repeatedly after already finding the reward within a trial; (3) Reference and working memory error: The animal repeatedly entered into arms without rewards. The total number of errors was evaluated as the overall spatial cognitive function index. This index was calculated by adding the number of “reference memory errors”, “working memory errors” and “reference and working memory errors”. Two rats (No. 7 in the control group, No. 17 in the MG group) in Phase II were excluded from the results of the test, because they made no attempt to seek the cereal despite appropriate weight restriction by limited feeding. 

### 4.4. Collection of Blood, Urine, Kidney and Brain Samples

Twenty-four-hour urinary samples were collected from the rats in a metabolic cage at the ages of 16 (Phase I) and 45 weeks (Phase II). All animals were sacrificed after the behavior tests were completed at the age 47 weeks. Pentobarbital (50 mg/kg) was administered intraperitoneally for euthanasia. Then the abdominal cavity was opened and as much blood as possible was collected from the descending aorta. Next, saline was perfused. After blood removal was completed, the bilateral kidneys were removed. Then the thorax was opened and perfused transcardially with phosphate-buffered saline (PBS) followed by 4% paraformaldehyde in 0.1 M phosphate buffer of pH 7.4. 

### 4.5. Sample Analysis

Methylglyoxal was measured using liquid chromatography/mass spectrometry, as previously reported [11].

In Phase I, we examined urinary 8-hydroxy-2'-deoxyguanosine (8-OHdG) and malondialdehyde (MDA). In addition to these two oxidative stress markers, we examined glutathione peroxidase (GPx) activity, superoxide dismutase (SOD) activity in the kidneys as an antioxidant marker, and angiotensinogen (AGT) in the urine and plasma were also examined in Phase II by a commercially available kit. We asked a laboratory testing company (SRL, Inc., Tokyo, Japan) to measure uric protein, uric and serum creatinine, blood urea nitrogen, plasma glucose and urinary 8-OHdG to. Urinary 8-OHdG is a marker of damage by DNA oxidation *in vitro*, which reflects the degree of oxidative stress [39]. MDA has been shown to be a major marker of lipid peroxidation [40]. We measured urinary MDA in both phases using a commercially available kit (Northwest Life Science Specialties LLC, Vancouver, WA, USA). The assay is based on the reaction of MDA with thiobarbituric acid (TBA2). After forming an MDA-TBA2 adduct, MDA was detected spectrophotometrically. The GPx assay kit is based on a method developed by Paglia *et al.* [41]. GPx activity was determined by following the oxidation of nicotinamide adenine dinucleotide phosphate (NADPH) at 340 nm in a glutathione reductase-coupled reaction, using hydrogen peroxide as the substrate [41]. Enzyme activity was defined as 1 µM of GSH conjugated/min at 25 °C [32]. The SOD activity assay in the kidney is based on a method of hematoxylin autoxidation developed by Martin *et al.* [42] (Northwest Life Science Specialties LLC, Vancouver, WA, USA). AGT was analyzed using the sandwich enzyme immunoassay method (Immuno-Biological Laboratories Co., Ltd., Gunma, Japan). 

Rat brain histological examination was conducted under light microscopy with hematoxylin and eosin (H & E) staining and caspase-3 staining. Fixed brains were cut into sections (6 µm thick) through the sagittal plane with a cryostat for H&E and caspase-3 staining. For caspase-3 staining, a rabbit anti-cleaved caspase-3 (Asp175) antibody (Cell Signaling Technology, Danvers, MA, USA) was used. Rat kidney histological examination was conducted under light microscopy with periodic acid-Schiff staining. Kidney samples obtained from each rat were fixed in 10% buffered formalin and embedded in paraffin and serially sectioned at 2.5 µm thickness. 

### 4.6. Statistical Analysis

Statistical analyses were performed using IBM SPSS Statistics version 19 (IBM, Armonk, NY, USA). All results were expressed as the mean ± standard error of the mean. Comparisons of means of the groups of the two behavior tests were analyzed by repeated one-way analysis of variance (ANOVA). After ANOVA, Turkey-Kramer’s *post hoc* test was used. Data analyses of the physical findings and laboratory tests, including serum MG and oxidative-stress or antioxidative markers and angiotensinogen (AGT), were performed using Student’s *t* test. Values of *p* < 0.05 were considered to indicate statistical significance. 

## 5. Conclusions

Long-term oral administration of MG in SD rats with normal kidney function did not cause CI, despite a significant increase in serum MG at the level of advanced CKD. Counter-balanced activation of the systemic anti-oxidant system by the MG load may offset the toxicity of MG in this model. Our results are considered to be important fundamental data for investigating MG toxicity under various conditions *in vitro*. The significance of MG in the pathogenesis of CI requires further investigation. 
